# COMBAR – Combatting anthelmintic resistance in ruminants[Fn FN1]

**DOI:** 10.1051/parasite/2023006

**Published:** 2023-02-10

**Authors:** Johannes Charlier, Hervé Hoste, Smaro Sotiraki

**Affiliations:** 1 Kreavet Hendrik Mertensstraat 17 9150 Kruibeke Belgium; 2 Université de Toulouse, UMR 1225 IHAP INRAE/ENVT 31076 Toulouse France; 3 Veterinary Research Institute, HAO DIMITRA Campus Thermi 57001 Thessaloniki Greece


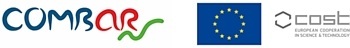


**Special Issue – Combatting Anthelmintic resistance in ruminants**

**Invited Editors: Johannes Charlier, Hervé Hoste, and Smaragda Sotiraki**

Cattle, sheep and goats are parasitized by various helminth species, the most important being the gastrointestinal nematodes (GIN) and liver fluke. These pathogens can cause severe disease and affect productivity in all classes of livestock and are worldwide amongst the most important production-limiting diseases of grazing ruminants [2]. Essentially, all herds/flocks in a grass-based production system are affected and the major economic impact is due to sub-clinical infections causing reduced growth and milk/wool production. These parasitic worms can also cause severe distress and disease, affecting animal welfare, and through the impact on farm management, production and food security, indirectly human wellbeing.

For more than 50 years, the control of these helminth infections has largely relied on the use of (broad spectrum) anthelmintics, belonging to different families of molecules. However, around the world, farmers and veterinarians are increasingly confronted with treatment failures due to anthelmintic resistance (AR). Frequent, indiscriminate or inappropriate use of anthelmintic drugs to control these parasites has resulted in selection of drug-resistant helminth populations. As shown in one of the papers in this special issue, AR in ruminants is now widespread for all the major GIN and is likely underreported in liver fluke [13]. AR has also been described in other GIN populations infecting host species like horses and pigs and is becoming a major concern for parasite control in companion animals and humans [18, 19]. If no changes to current control procedures are made, the health and welfare of animals and people are expected to be seriously impacted. In parallel, this is expected to lead to major economic losses and impacts regarding food security and greenhouse gas emissions from livestock through efficiency losses. This is particularly so in specific production systems like grazing dairy sheep and goats, where regulations to avoid medicine residues in milk have limited the available anthelmintic compounds. This has likely accelerated the development of AR to the few available molecules [14].

Previous research projects had attempted to tackle AR nationally or via projects focused on a specific component of the AR conundrum. These had led to limited progress in several areas including diagnostics to detect AR, socio-economic aspects and integration of the different control tools. The COST Action COMBAR therefore came at a time of high need to coordinate research efforts and exchange approaches at the European and international level. The main aim of COMBAR (Combatting Anthelmintic Resistance in Ruminants; https://www.combar-ca.eu) was to create a network and foster the exchange of scientific knowledge required to COMBAT AR and bring recognised experts in the field of sustainable control of helminth parasites in livestock, from across Europe and beyond, together. An important achievement has been the development of the STAR-IDAZ IRC research road maps on helminths and AR, offering a framework to coordinate research in the field at a global level and speed up the delivery of required control tools [15]. The outcomes of this exercise have also been summarised in a COMBAR document on the key priority research needs [3]. Another important output is recommendations for decisions makers to achieve sustainable helminth control [6]. By the end of the action, over 200 scientists from 31 COST member countries as well as other countries (USA, Mexico, Canada, Brazil, South Africa, and New Zealand) participated in one or more activities enhancing global knowledge exchange on the topic.

COMBAR was organised according to three working groups: (i) Improving diagnosis; (ii) Socio-economic aspects, and (iii) Innovative control approaches. This special issue in *Parasite* aims at presenting a range of articles underlining the different fields of activities and collaboration dynamics within COMBAR. It illustrates the diversity of scientific disciplines (e.g., parasitology, infectiology, epidemiology, molecular biology, phytochemistry, and socio-psychology) and institutions involved. The development of resistance to synthetic molecules is a general phenomenon which has been described for a wide range of pathogens and molecules [20]. Within this frame, the articles presented here also have more generic implications for resistance to antiparasitics in other animal species as well as in humans.

A major effort was made by Vineer et al., who created an open database summarising 535 AR surveys in 22 countries to represent the most up to date situation on the prevalence of AR in sheep, goats and cattle in Europe [13]. Knowing where the parasites are can underpin regionally targeted control measures. Thus, Hendrickx et al. used historical data to compare species distribution models detecting “hot spots” of the lancet fluke *Dicrocoelium dendriticum* [5]. They concluded that an even spatial distribution of the input data is more important than the actual sample size, hence the importance of correcting for sample biases when data originally collected for other purposes are used. The study of Untersweg et al. found reduced anthelmintic efficacy against different parasite species in a majority of examined sheep flocks in Austria and flags the rise of *Haemonchus contortus* infections in areas where they were previously rarely encountered [16]. Gravdal et al., on the other hand, conducted a survey among >5000 sheep farmers in Norway and concluded that, as parasitological analysis is seldomly performed on farm, most farmers were unaware of the parasite species or infection levels present on their farm [4]. Both studies point to a need for greater engagement of farmers and veterinarians in sustainable parasite control. The fact that such engagement may yield significant economic benefits was shown by Martínez-Valladares et al. who found a negative correlation between the pre-partum faecal egg count (FEC) and subsequent milk production, as well as a significant difference in milk yield between flocks that received anthelmintic treatment or not [11]. Sustainable helminth control requires the availability of reliable and cost-effective diagnostics to assess infection levels as well as the presence of AR. Babják et al. found that the *in vitro* egg hatch test can accurately estimate *in vivo* efficacy following treatment of *H. contortus* infections with benzimidazoles in goats and that it also reflects the percentage of resistance alleles in the parasite population [1]. Khangembam et al. present an optimised LAMP assay for the detection of *H. contortus* in small ruminant faecal samples [9]. Both studies are an important step towards quicker and more cost-effective diagnosis of GIN infections (in this case *H. contortus*) and their resistance status. Today, on farm diagnosis of anthelmintic efficacy against all nematode species is based on assessing FEC reduction following treatment. Morgan et al. evaluate the wide range of factors that can confound such a diagnostic approach and recommend how this can be taken into account to enhance correct interpretation [12]. Diagnostics are only of value if they allow us to adopt sustainable control practices. In this special issue, we therefore also report on the evaluation of complementary control approaches, such as plant-based control that may reduce the need for chemotherapy. Hoste et al. identify the use of tannin containing agroindustrial by-products as new resources for antiparasitic forages and compounds, highlighting the move towards environmentally friendly and circular animal health approaches [7]. Ježek et al. identified the use of pumpkin seed cakes and cloves as a promising feed additive, leading to lower FECs in sheep [8]. Maestrini et al., on the other hand, showed that aqueous extracts of liquorice roots may present a promising substance for further development as a natural dewormer [10].

The results reported in this COMBAR special issue represent a very small step for the new tools and approaches that are needed to combat AR. Nonetheless, we hope they will encourage the reader to continue along the path to sustainable helminth control. Besides the continuous needs for fundamental research to understand the mechanisms of AR, there is also a very large need for applied research to develop sustainable control methods with the current tools at hand. Finally, as shown by the work of Vande Velde et al. [17], communication and communication research is essential for the correct and widespread implementation of any sustainable control approach. Therefore, different communication strategies are likely required in different countries, making use of a mixture of targeted and region-specific messages, involvement of innovator and early adaptor farmers, as well as nudging approaches.

We would like to thank all contributing authors, reviewers and the COMBAR Core Group members for their important contributions and making this special issue possible. Special thanks go to Jean-Lou Justine, Editor-In-Chief of *Parasite*, for his unwavering support. Finally, a big thank you to all COMBAR participants for their contributions that enabled us to leave a strong COMBAR legacy for the future.
